# Insights on the Inhibitory Power of Flavonoids on Tyrosinase Activity: A Survey from 2016 to 2021

**DOI:** 10.3390/molecules26247546

**Published:** 2021-12-13

**Authors:** Heba A. S. El-Nashar, Mariam I. Gamal El-Din, Lucian Hritcu, Omayma A. Eldahshan

**Affiliations:** 1 Department of Pharmacognosy, Faculty of Pharmacy, Ain Shams University, Abbassia, Cairo 11566, Egypt; heba_pharma@pharma.asu.edu.eg (H.A.S.E.-N.); dr_mariam_gamal_eldin@pharma.asu.edu.eg (M.I.G.E.-D.); 2 Department of Biology, Faculty of Biology, Alexandru Ioan Cuza University of Iasi, Bd. Carol I, No. 11, 700505 Iasi, Romania; 3 Center of Drug Discovery Research and Development, Faculty of Pharmacy, Ain Shams University, Abbassia, Cairo 11566, Egypt

**Keywords:** tyrosinase, melanin, skin, aging, flavonoids, antioxidant

## Abstract

Tyrosinase is a multifunctional copper-containing oxidase enzyme that initiates melanin synthesis in humans. Excessive accumulation of melanin pigments or the overexpression of tyrosinase may result in skin-related disorders such as aging spots, wrinkles, melasma, freckles, lentigo, ephelides, nevus, browning and melanoma. Nature expresses itself through the plants as a source of phytochemicals with diverse biological properties. Among these bioactive compounds, flavonoids represent a huge natural class with different categories such as flavones, flavonols, isoflavones, flavan-3-ols, flavanones and chalcones that display antioxidant and tyrosinase inhibitor activities with a diversity of mechanistic approaches. In this review, we explore the role of novel or known flavonoids isolated from different plant species and their participation as tyrosinase inhibitors reported in the last five years from 2016 to 2021. We also discuss the mechanistic approaches through the different studies carried out on these compounds, including in vitro, in vivo and in silico computational research. Information was obtained from Google Scholar, PubMed, and Science Direct. We hope that the updated comprehensive data presented in this review will help researchers to develop new safe, efficacious, and effective drug or skin care products for the prevention of and/or protection against skin-aging disorders.

## 1. Introduction

Tyrosinase is a multifunctional copper-containing oxidase enzyme, involved in the initial steps of melanin production [1]. Melanin synthesis is regulated by the microphthalmia-associated transcription factor (MITF) that is activated by multiple pathways such as cAMP-responsive element-binding protein (CREB), Wnt, glycogen synthase kinase 3β, and mitogen-activated protein kinases, and in turn modulates melanogenic enzyme expression such as tyrosinase [2]. This enzyme catalyzes the oxidation of L-tyrosine or L-3,4-dihydroxyphenylalanine (L-DOPA) to DOPA-quinone, which is the rate-limiting step of melanin synthesis [3].

Excessive production of melanin pigments resulted in different dermatological disorders such as skin aging spots, wrinkles, melasma, freckles, lentigo, ephelides, nevus and melanoma [4]. More seriously, tyrosinase catalyzes the oxidation of dopamine into quinone derivatives that initiate the progress of Parkinson disease [5]. These physiological disturbances are primarily ascribed to the excess accumulation of reactive oxygen species (ROS) or oxidative stress, which can affect the biological macromolecules and cellular functions, thus leading to aging-related disorders or hyperpigmentation [6]. Many patients suffer from these ailments, which have a damaging effect on their quality of life [7]. Furthermore, currently available hypopigmenting agents, such as hydroquinone or kojic acid, have serious side effects such as carcinogenesis, hepatotoxicity, and dermatitis [7]. Therefore, the discovery of antioxidants and anti-tyrosinase drugs that inhibit tyrosinase catalytic activity or modulate its expression has been a widely recognized approach in the field of skin care products and pharmaceuticals for protection against skin aging, wrinkling, and browning [8].

Natural products from plants have been used for centuries as a rich resource for curing various ailments. They provide unique structural diversity in comparison to standard combinatorial chemistry, which presents opportunities for discovering novel low molecular-weight lead compounds [9]. Several medicinal plants have been reported for tyrosinase inhibitory effects such as *Asphodelus microcarpus*, *Morus nigra* L. *Limonium tetragonum*, *Arctostaphylos uva-ursi*, *Artocarpus lowii*, *Artemisia aucheri*, *Cassia tora*, *Silybum marianum*, *Humulus lupulus*, *Rhodiola crenulata*, *Alpinia officinarum*, *Mangifera indica*, *Podocarpus falcatus*, *Momordica charantia*, *Cymbopogon citrates*, *Vitis vinifera* and *Glycyrrhiza glabra* [10].

Flavonoids are major polyphenolic groups of the plant kingdom, widely distributed in fruits, vegetables, cereals, and beverages we consume daily [11]. They are categorized according to the hydroxylation and saturation pattern of heterocyclic C-ring structure into flavones, flavonols, isoflavones, flavan-3-ols, flavanones and chalcones [12]. Flavonoids possess strong antioxidant properties due to their ability to stabilize free radicals or ROS by donating a hydrogen atom or single-electron transfer [13]. Additionally, they could effectively induce the production of internal antioxidant defensive enzymes such as UDP-glucuronosyltransferases, sulfotransferases, N-acetyltransferases, glutathione *S*-transferases, superoxide dismutase, catalase, and methyltransferases [14]. In addition, they act as chelators for metals due to their characteristic polyhydroxylated phenolic structure, which can incorporate copper ions of the tyrosinase active site [15]. Furthermore, flavonoids have received much attention from scientists recently due to their low toxicity on tyrosinase [3]. Recent studies have reported the importance of flavonoids as promising whitening models via the inhibition of tyrosinase enzyme, thereby controlling melanin production [16]. In this review, we focus on the role of novel or known flavonoids isolated from different plant species and their participation as tyrosinase inhibitors reported in the last five years from 2016 to 2021. We also discuss the mechanistic approaches through different studies carried out on these compounds including in vitro, in vivo and in silico computational research. We hope that the updated comprehensive data presented in this review will help researchers to develop new safe, effective drug or skin care products for the prevention and protection against skin-aging disorders.

## 2. The Role of Tyrosinase in the Pathway of Melanin Biosynthesis

Tyrosinase is bio-functional copper-containing enzyme implicated in multidisciplinary functions such as browning in plants, skin melanogenesis cascade in humans, the differentiation of the reproductive system, the host defense system in arthropods, and spore development in fungi [17]. Tyrosinase is mainly known as the key enzyme that regulates the quantity of melanin pigment formation in mammals [18]. Melanin is found in two major forms of pigments, namely eumelanin (black-brownish) and pheomelanin (red-yellowish) [19]. The quantity and combination of these two forms can influence the colour of the skin, eyes, and hair. Furthermore, these pigments protect the human skin against harmful UV radiation and prevent DNA mutations and the progression of skin cancer. On other hand, the overproduction of melanin pigments could lead to hyperpigmentation diseases, such as lentigo, ephelides, melasma, freckles and nevus [20].

**Figure 1:**
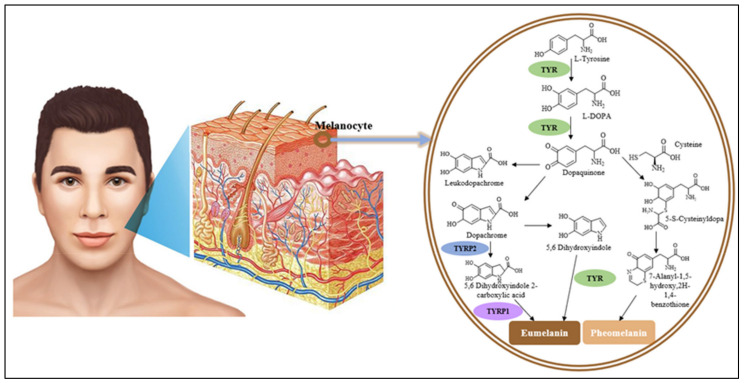
The pathway of melanin biosynthesis catalyzed by tyrosinase. TYR, tyrosinase; TYRP2, tyrosinase-related protein-2; TYRP1, tyrosinase-related protein-1.

When a human is exposed to ultra-violet (UV) light, tyrosinase is activated and inductively proceeded in several intermediate phases of skin pigmentation [21]. Tyrosinase can activate two different reactions: hydroxylation of tyrosine to L-dopa, followed by oxidation to o-quinones (dopaquinone, catecholase) [22]. Then, dopaquinone is spontaneously converted to dopachrome, dopachrome tautomerase (TRP2/DCT, tyrosinase-related protein-2), which directly transforms into 5,6-dihydroxyindole-2-carboxylic acid (DHICA). After that, DHICA oxidase (TRP-1, tyrosinase-related protein-1) catalyzes the oxidation of DHICA into indole-quinone-carboxylic acid [23,24]. As shown in Figure 1, tyrosinase-related proteins including TRP-2 and TRP-1 could control the end steps of eumelanin bioconversion, and thereby the type of synthesized eumelanin. Additionally, tyrosinase proteins regulate the biological effects of melanocytes in certain ways [25].

## 3. Flavonoids with Promising Anti-Tyrosinase Activities

### 3.1. Flavones 

An antioxidant flavonoid, namely tricin (3′,5′-dimethoxy-4′,5,7-trihydroxyflavone) (**1**), was reported in the ethyl acetate extract of rice husk [26]. It showed an IC_50_ of 0.312 mg/mL by means of the 2,2′-azino-bis (ABTS) assay, compared with butylated hydroxytoluene (BHT) (IC_50_ = 0.08 mg/mL). Furthermore, tricin showed β-carotene bleaching of about 76.07%, compared to BHT (86.66%). Interestingly, this compound inhibited tyrosinase by 15.69%, comparable with vanillin (inhibition% = 13.24%) at the concertation of 2 mg/mL. Luteolin-7-sulfate (3′,4′,5-trihydroxy-flavone-7-sulphate) (**2**), an uncommon flavonoid, was isolated from a few plants such as *Phyllospadix iwatensis* and *Zostera marina* [27]. This compound potently suppressed melanin synthesis by ten times more than arbutin via the regulation of MITF, tyrosinase expression and CREB signaling pathways in murine melanoma B16-F10 cells [3]. Additionally, luteolin-7-sulfate (**2**) dose-dependently suppressed melanin synthesis in primary human epidermal melanocytes (HEMs). On the other hand, luteolin 7-sulfate exerted lower toxicity compared to luteolin in B16-F10 cells. Ticona et al. [28] isolated four anti-tyrosinase flavonoids from dichloromethane extract of the aerial parts of *Loranthus acutifolius*. These compounds were identified as 2′,4′,6-trimethoxyflavone (**3**) (IC_50_ = 4.00 ± 0.03 μM), 3′,4′,5-trihydroxy-6,7,8-trimethoxyflavone (**4**) (IC_50_ = 11.30 ± 0.05 μM), 2′4′-dihydroxy-6′-methoxy-chalcone (**56**) (IC_50_ = 5.70 ± 0.02 μM) and 4′,5-dihydroxy-6,7,8-trimethoxyflavone (**5**) (IC_50_ = 8.60 ± 0.04 μM), comparable with kojic acid (IC_50_ = 13.90 ± 0.34 μM). Furthermore, they suppressed melanin content in B16-F10 murine melanoma cells with IC_50_ values ranging from 1.60 ± 0.03 to 8.10 ± 0.05 μM, while kojic acid showed an IC_50_ =13.90 ± 0.34 μM. Interestingly, 2′,4′,6-trimethoxyflavone (**3**) exerted the most remarkable anti-melanogenic potential, attributed to its structural similarity with tyrosine amino acid that makes it fit as a competitive substrate on tyrosinase.

Tentative phytochemical research was conducted on the 50% methanol extract of *Oroxylum indicum* (Bignoniaceae) seeds using HPLC/TOF-MS, and then the identified compounds were screened for tyrosinase-binding affinity [29]. Among these compounds, two flavonoids, namely baicalein (5,6,7-trihydroxyflavone) (**6**) and oroxin A (baicalin-7-glucoside) (**7**), exhibited the most potent inhibitory activities against the enzyme, with inhibition values of 64.90% and 59.70%, and IC_50_ values of 0.29 and 0.50 mM. The molecular docking assays attributed these remarkable anti-tyrosinase properties to the structural similarities that make them fit on the same amino acid residues in the TYR catalytic pocket. Furthermore, the glycosylation of flavonoids could reduce their inhibitory activity.

Isovitexin (**8**), apigenin-6-C-glucoside, was isolated from the methanol extract of *Achillea alpina* L.’s aerial parts [30]. It effectively suppressed melanin synthesis via the down-regulation of intracellular tyrosinase signaling. Based on the structure–activity relationship, the presence of the hydroxyl group at the A and B rings is essential for the inhibitory activity. A certain study was conducted on twenty antioxidant phenolic compounds isolated from *Trichosanthes kirilowii* fruits [31]. Among the screened compounds, diosmetin (3′,5,7-trihydroxy-4′-methoxyflavone) (**9**), chrysoeriol (4′,5,7-trihydroxy-3′-methoxyflavone) (**10**), scutellarin (4′,5,6,7-tetrahydroxy-flavone-7-*β*-d-glucopyranuronoside (**11**) and 3′,5-dihydroxy-7-(*β*-d-glucopyranosyloxy)−4′-methoxyflavone (**12**) significantly concentration-dependently inhibited tyrosinase activity and melanin production, compared to arbutin as a positive control. The authors reported that the predominance of hydroxyl groups in the B ring could enhance the inhibitory effect. Sulfuretin (6,3′,4′-trihydroxyaurone) (**13**) is a predominant flavonoid of *Rhus verniciflua* (Anacardiaceae), and is known to exhibit antioxidant activity [32]. This compound exhibited a direct inhibitory effect on tyrosinase activity (IC_50_ = 20 μM), compared with kojic acid. In vivo studies showed its ability to inhibit cellular melanogenesis in neonatal human melanocytes and melanoma B16 cells treated with forskolin or α-MSH. Furthermore, it significantly suppressed melanin synthesis through the downregulation of cAMP and MITF expression in human primary melanocytes. Artocaepin E (**14**), isolated from the wood of *Artocarpus heterophyllous* with other seven compounds, was proved to possess promising potent tyrosinase inhibitory effect, with an IC_50_ of 6.7 ± 0.8 μM. This flavone demonstrated competitive inhibition on tyrosinase enzyme with an inhibition constant (*K*i) of 6.23 μM. [33].

Genkwanin (4′,5-Dihydroxy-7-methoxyflavone) (**15**), previously isolated from *Daphne gnidium* stems, Alnus glutinosa seeds, and Asplenium normale leaves, was evaluated for its tyrosinase activity and its effect on melanin synthesis [34]. It demonstrated dose- and time-dependent inhibition of tyrosinase activity in B16F10 melanoma cells. It also caused a significant dose-dependent reduction in melanin synthesis. Apigenin-7-*O*-*β*-d-glucopyranoside (**16**), the abundant flavone in different *Thymus* species, was evaluated at different concentrations for its tyrosinase activity and its influence on melanin synthesis [35]. It demonstrated significant stimulation of tyrosinase activity of B16F10 melanoma cells in a dose and time dependent manner. In addition, the flavone dose-dependently stimulated the synthesis of intracellular melanin. The up-regulation of the expression and the activity of the microphthalmia-associated transcription factor regulating the gene transcription of tyrosinase as well as activation of the cyclic AMP-protein kinase A pathway were the supposed underlying mechanisms. In a comparative study of the tyrosinase-inhibitory activities of fifty flavonoids, the antioxidant flavone swertiajaponin demonstrated the most potent inhibitory activity against mushroom tyrosinase, with an IC_50_ value of 43.47 μM, comparable to the positive control kojic acid, with an IC_50_ value of 41.26 μM [36]. Swertiajaponin (6-C-*β*-d-glucopyranosyl-7-*O*-methylluteolin) (**17**), abundant in *Swertia japonica* and *Cymbopogon citratus*, demonstrated significant inhibition of skin pigmentation in a human skin model in addition to the depression of melanin accumulation in αMSH- or UVB-induced B16F10 cells. The interaction with the binding site of tyrosinase enzyme in addition to the reduction in tyrosinase protein levels via the inhibition of stress-mediated MAPK/MITF signaling is the suggested underlying mechanism behind the potent tyrosinase-inhibiting activity.

**Figure 2:**
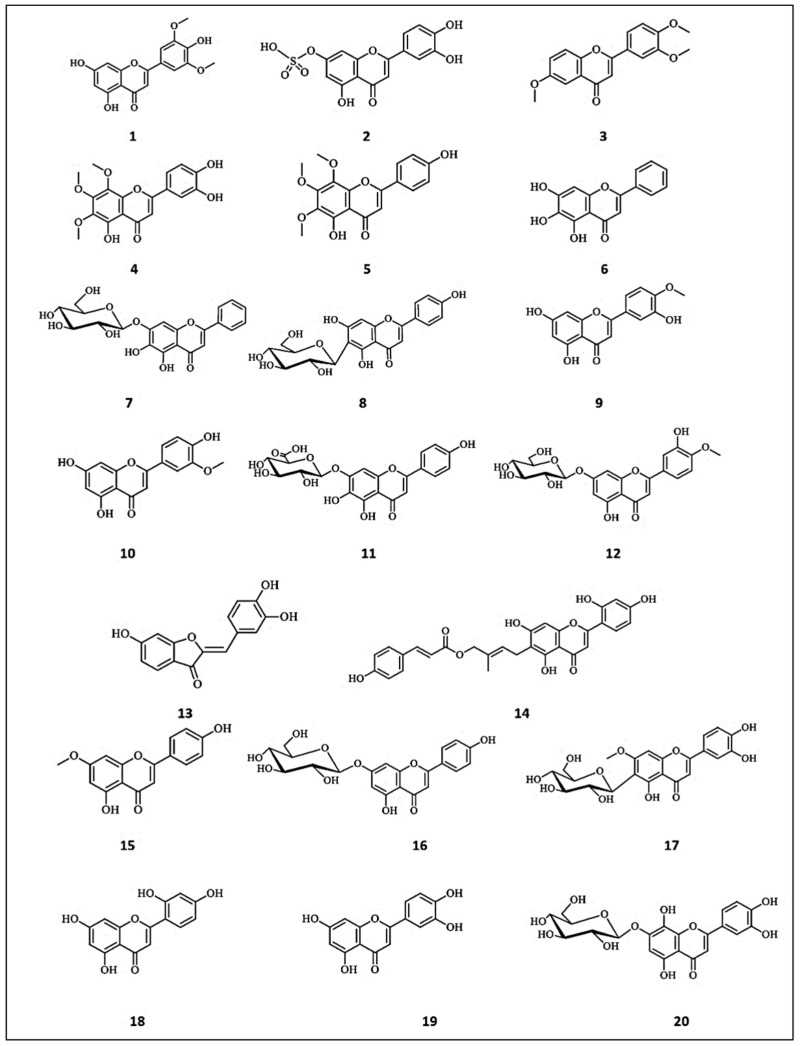
The chemical structures of flavones with anti-tyrosinase activity. (**1**) Tricin; (**2**) Luteolin-7-sulfate; (**3**) 2′,4′,6-trimethoxyflavone; (**4**) 3′,4′,5-trihydroxy-6,7,8-trimethoxyflavone; (**5**) 4′,5-dihydroxy-6,7,8-trimethoxyflavone; (**6**) Baicalein; (**7**) Oroxin A; (**8**) Isovitexin; (**9**) Diosmetin; (**10**) Chrysoeriol; (**11**) Scutellarin; (**12**) 3′,5-dihydroxy-7-(β-d-glucopyranosyloxy)−4′-methoxyflavone; (**13**) Sulfuretin; (**14**) Artocaepin E; (**15**) Genkwanin; (**16**) Apigenin-7-*O*-*β*-d-glucopyranoside; (**17**) Swertiajaponin; (**18**) Norartocarpetin; (**19**) Luteolin; (**20**) Hypolaetin-7-*O*-β-d-glucopyranoside.

A comparative study was conducted for tyrosine inhibitory activities of structurally related flavones; norartocarpetin (5,7,2′,4′-tetrahydroxyflavone) (**18**) and luteolin (3′,4′,5,7-tetrahydroxy-flavone) (**19**) [37]. Despite the great similarity in the chemical structures of both flavones differing only in the position of a hydroxyl group, a vast difference was observed in their tyrosinase inhibitory activities. Norartocarpetin (**18**) demonstrated nearly 2200-fold stronger tyrosinase inhibitory activity than luteolin, with IC_50_ values of 0.12 μM compared with luteolin (IC_50_ = 266.67 μM). In addition, norartocarpetin demonstrated 570-fold stronger tyrosinase activity than kojic acid (IC_50_ = 266.67 μM). The kinetic studies demonstrated the strong reversible competitive inhibition of tyrosine induced by norartocarpetin. However, luteolin (**19**) demonstrated weak, noncompetitive reversible inhibition. Computational docking simulations explained the reversible competitive inhibition induced by norartocarpetin by its ring B hydroxyl groups binding to Asn81 and His85 residues located in the catalytic pocket of tyrosinase. Meanwhile, the hydroxyl groups of the B ring of luteolin bind to residues Cys83 and Asn8. In another study, Park et al. [38] isolated fourteen flavonoids from the fruits of *Juniperus chinensis* and evaluated their inhibitory activity on mushroom tyrosinase. Among these compounds, hypolaetin-7-*O*-*β*-d-glucopyranoside (8-hydroxyluteolin-7-*O*-*β*-d-glucopyranoside) (**20**) and quercetin-7-*O*-*α*-l-rhamnopyranoside (**21**) were found to reduce tyrosinase activity at a concentration of 50 μM. They could effectively attenuate the cellular tyrosinase activity and melanogenesis in α-MSH plus IBMX-stimulated B16F10 melanoma cells. The chemical structures of the flavones with anti-tyrosinase activities are presented in Figure 2.

### 3.2. Flavonols

Molecular docking simulation studies revealed that quercetin-7-*O*-*α*-l-rhamnpyranoside (**21**) inhibited the enzyme by hydrogen bonding with residues His85, His244, Thr261, and Gly281 of tyrosinase. In addition, quercetin-7-*O*-*α*-l-rhamnoside (**21**) demonstrated the most potent inhibitory activity on mushroom tyrosinase (56.75 μM) among the isolated flavonoids [38]. Fractionation of a 95% ethanol extract from *Citrus sinensis* peel yielded petroleum ether, ethyl acetate and water extracts [39]. Among these extracts, the ethyl acetate fraction showed the most potent inhibitory effect on tyrosinase with IC_50_ value of 108.24 μg/mL. Interestingly, phytochemical investigation afforded three flavonols, namely sinensetin (**22**), 4′,5,6,7-tetramethoxyflavone (**23**), nobiletin (**24**) and 3,3′,4′,5,6,7-hexamethoxyflavone (**25**).

*Rosa rugosa* extract (Rosaceae) is rich in polyphenolic compounds with anti-tyrosinase activity [40]. The isolated flavonoid compounds, namely hyperoside (**26**), kaempferol-3-*O*-rutinoside (**27**), and rutin (quercetin 3-rutinoside) (**28**), exhibited potent inhibitory activity (IC_50_ ˂ 1 μg/mL) on mushroom tyrosinase compared to the standard drug, kojic acid (80.00 μg/mL). Another phytochemical study was conducted on the methanol extract of *Myrsine africana* (Myrsinaceae) shoots, resulting in the isolation of twelve flavonoid glycosides [41]. Among these compounds, rutin (**28**) and myricetin-3-*O*-*α*-l-rhamnopyranoside (**29**) exerted the most promising tyrosinase inhibitory effects with IC_50_ values of 0.13 ± 0.003 and 0.12 ± 0.002 mM, respectively, compared to kojic acid (0.01 ± 0.001 mM). Moreover, both compounds had remarkable antioxidant properties with IC_50_ of 2.30 ± 0.002 and 2.00 ± 0.006 μM, which were more potent than ascorbic acid (positive control; IC_50_= 11.20 ± 1.36 μM) using the DDPH assay. Two flavonoid glycosides; myricetin-3-*O*-*β*-galactopyranoside (**30**) and quercetin-3-*O*-*β*-galactopyranoside (**31**) were identified as tyrosinase inhibitors from *Limonium tetragonum* in B16-F10 cells [42]. Furthermore, they displayed anti-melanogenic capacities with an efficiency superior to that of kojic acid, attributing the downregulation of microphthalmia-associated transcription factor, tyrosinase-related protein-1, and tyrosinase-related protein-2 at the mRNA and protein expression levels. Both glycosides demonstrated inhibition of cellular tyrosinase activity by 65% and 63%, respectively, relative to 59% inhibition of tyrosine activity by kojic acid. In addition, they significantly reduced the protein levels of tyrosinase-related protein-1 and 2 (TRP-1 and TRP-2) with an efficiency exceeding that of kojic acid. The underlying mechanisms of potent tyrosine inhibition of flavonol glycosides were suggested to be through their involvement in regulating tyrosinase activity through TRP-linked pathways in addition to their antioxidant properties [42].

In a comparative study of the tyrosine inhibitory activity of nine flavonols and flavonol glycosides, quercetin-3-*O*-*β*-galactopyranoside (**31**) demonstrated the most potent significant inhibitory activity (IC_50_ = 40.94 ± 0.78 µM) [43]. An in silico study supported the results demonstrating the crucial value of the hydroxyl group at the 7th position for competitive inhibition of tyrosinase enzyme. Moreover, the study supported the importance of hydroxyl substitution at C-3′ and C-4′ in binding with tyrosinase enzyme.

A certain in vitro study was conducted on the aerial parts of *Cotula anthemoides* L. (Asteraceae), resulting in the isolation of a new sulfonyl flavonol glucoside, namely 5,7,4′,5′-tetrahydoxyflavonol 2′-[propanoic acid-(2′′′-acetoxy-1′′′-sulfonyl)]−5′-*O*-*β*-d-glucopyranoside (**32**) [44]. This compound showed moderate inhibitory effect on tyrosinase enzyme (IC_50_ = 100 ± 0.5 μM), comparable to kojic acid (6.4 ± 0.04 μM). Quercetin (**33**), the most abundant flavonol in green tea, apples, berries, onions, and many other fruits, vegetables and grains was proved to possess potent inhibitory activity on the monophenolase and diphenolase activities of tyrosinase. An in silico study by Fan et al. [45], demonstrated (**33**) the reversible and competitive inhibition of quercetin on the diphenolase activity of tyrosinase with an IC_50_ value of (3.08 ± 0.74) × 10^−5^ mol L^−1^. The interaction of the 3′, 4′-dihydroxy groups of quercetin (**33**) with copper ions at the active sites is beyond the inhibitory activity of quercetin (**33**), as revealed by molecular docking studies. In another study with quercetin (**33**) isolated from *Persicaria senticosa* (Polygonaceae), it was reported to inhibit tyrosinase with IC_50_ values of 14.31 ± 3.93 μM, which is close to the positive control (kojic acid; IC_50_ = 11.38 ± 4.16 μM) [46]. Moreover, it showed a potent DPPH-scavenging property (IC_50_ = 22.71 ± 0.53 μM), comparable to BHA (IC_50_ = 271.70 ± 19.45 μM).

**Figure 3:**
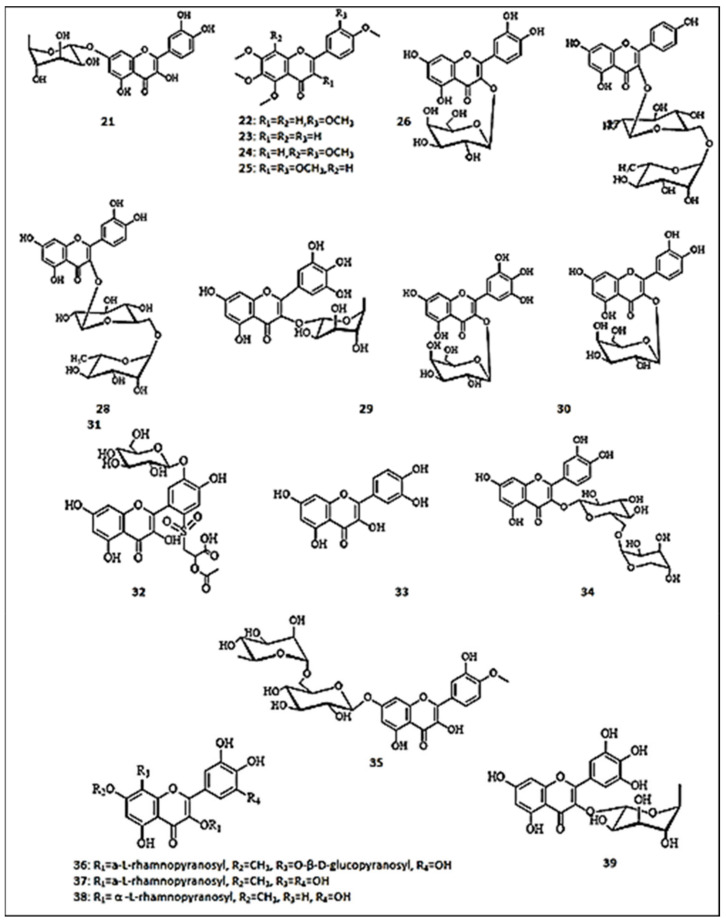
The chemical structures of flavonols with anti-tyrosinase activity. (**21**) Quercetin-7-*O*-α-l-rhamnpyranoside; (**22**) Sinensetin; (**23**) 4′,5,6,7-tetramethoxyflavone; (**24**) Nobiletin; (**25**) 3,3′,4′,5,6,7-hexamethoxyflavone; (**26**) Hyperoside; (**27**) Kaempferol-3-*O*-rutinoside; (**28**) Rutin; (**29**) Myricetin-3-*O*-α-l-rhamnopyranoside; (**30**) Myricetin-3-*O*-β-galactopyranoside; (**31**) Quercetin-3-*O*-β-galactopyranoside; (**32**) 5,7,4′,5′-tetrahydoxyflavonol 2′-[propanoic acid-(2″’-acetoxy-1″’-sulfonyl)]−5′-*O*-β-d-glucopyranoside; (**33**) Quercetin; (**34**) Quercetin-3-*O*-α-arabinopyranosyl-(1→6)-β-glucopyranoside; (**35**) Hesperidin; (**36**) 8-β-d-glucopyranosyloxy-3′,4′,5,5′-tetrahydroxy-7-methoxy-3-α-l-rhamnopyranosyloxyflavone; (**37**) 3′,4′,5,5′,8-pentahydroxy-7-methoxy-3-α-l-rhamnopyranosyloxyflavone; (**38**) Europetin 3-*O*-α-l-rhamnopyranoside; (**39**) Myricetin-3-*O*-α-l-rhamnopyranoside.

A molecular modeling study evaluated the anti-tyrosinase activity of nine structurally related flavonols, naturally occurring in many plants [16]. Among the tested compounds, quercetin-3-*O*-*α*-arabinopyranosyl-(1→6)-*β*-glucopyranoside (**34**) inhibited the enzyme with an IC_50_ value of 46.94 ± 3.09, near that of kojic acid (IC_50_ = 45.69 ± 1.65 µM). Similarly, the methanol extract of *Scrophularia lucida*’s aerial parts yielded hesperidin (hesperetin-7-rutinoside) (**35**) (394 μg/g extract) as a major component [47]. This compound exhibited high docking value for tyrosinase enzyme. In another in silico study, more than thirty antioxidant phenolic compounds were isolated from the aerial parts of *Atraphaxis frutescens* and investigated for their inhibitory actions on mushroom tyrosinase [48]. It was observed that four new 7-methoxyflavonoids demonstrated anti-tyrosinase effects, such as 8-*β*-d-glucopyranosyloxy-3′,4′,5,5′-tetrahydroxy-7-methoxy-3-*α*-l-rhamnopyranosyloxyflavone (**36**) (IC_50_= 0.90 ±0.02 mM), 3′,4′,5,5′,8-pentahydroxy-7-methoxy-3-*α*-l-rhamnopyranosyloxyflavone (**37**) (IC_50_ =1.20 ± 0.03 mM) and europetin 3-*O*-*α*-l-rhamnopyranoside (**38**) (IC_50_ =1.10 ± 0.07 mM), compared to kojic acid (IC_50_ = 0.088 ± 0.002 mM). However, isolated flavonol aglycones did not show any inhibitory properties. Hence, the presence of 3-*O*-rhamnosyl moiety was suggested to participate in tyrosinase inhibition. Kishore et al. investigated the tyrosine inhibitory activity of myricetin-3-*O*-*α*-l-rhamnopyranoside (**39**), isolated from *Myrsine africana* methanol extract [41]. It demonstrated significant inhibitory activity on tyrosinase, demonstrating a IC_50_ value of 0.12 ± 0.002 mM. The molecular docking analysis supported its inhibitory activity. The chemical structures of the flavonols with anti-tyrosinase activities are presented in Figure 3.

### 3.3. Isoflavones 

**Figure 4:**
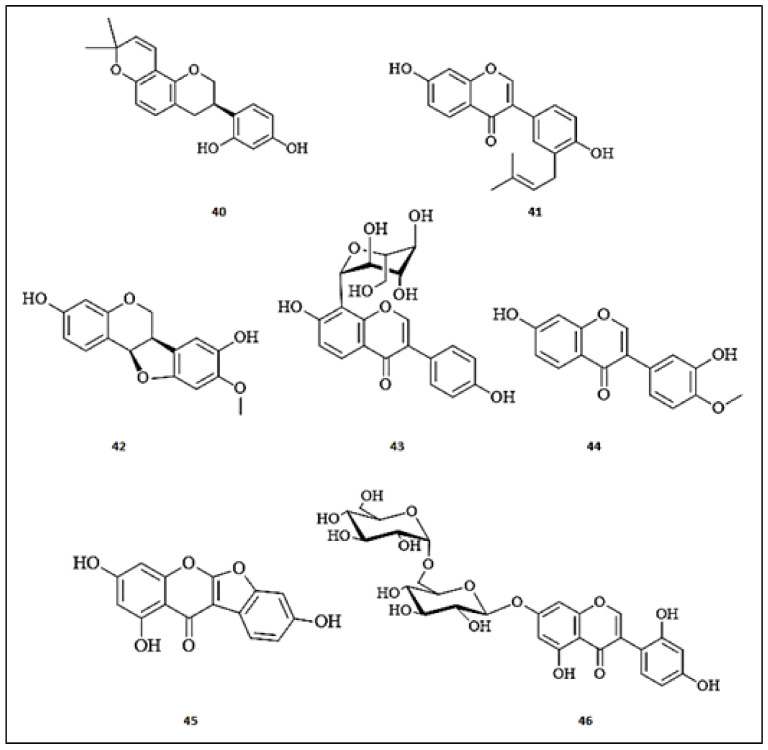
The chemical structures of isoflavones with anti-tyrosinase activity. (**40**) Glabridin; (**41**) Neobavaisoflavone; (**42**) (6aR,11aR)-3,8-dihydroxy-9-methoxy pterocarpan; (**43**) Puerarin; (**44**) Calycosin; (**45**) Lupinalbin A; (**46**) 2′-hydroxygenistein-7-*O*-gentibioside.

Glabridin (**40**), an isoflavanoid isolated from *Glycyrrhiza glabra* roots, was investigated for inhibitory effects and binding mechanisms on tyrosinase [49,50]. This compound non-competitively inhibited tyrosinase with an IC_50_ value of 0.43 μmol/L through a multiphase kinetic process. Further, glabridin (**40**) forms stable a glabridin-tyrosinase complex through the strong quenching of intrinsic tyrosinase fluorescence. The molecular docking studies confirmed non-direct binding of glabridin (**40**) to the tyrosinase active site. Moreover, glabridin (**39**) did not exert any side effects on melanin synthesis or toxicity in a zebrafish model [50]. Kim et al. identified a prenylated isoflavonoid, neobavaisoflavone (**41**) from the ethanolic extract of the aerial parts of *Pueraria 1lobate* (Fabaceae) [51]. This compound (**41**) significantly reduced the mRNA and protein expression levels of MITF, TRP-1, and tyrosinase in α-MSH-stimulated B16F10 murine melanoma cells. It potently inhibited tyrosinase activity at a rate of 45.50% at 50 µM, compared to arbutin (12%). Additionally, it effectively reduced melanin content in a reconstructed human 3D skin model, compared to arbutin. Mechanistically, it suppressed the melanin production through modulation of Akt/GSK−3β and MEK/ERK signaling transduction pathways. About twenty-seven flavonoids were identified by Promden et al. [52] in *Dalbergia parviflora* and tested for tyrosinase inhibition. Among these flavonoids, (6aR,11aR)-3,8-dihydroxy-9-methoxy pterocarpan (**42**) was the most promising tyrosinase inhibitor by 84.60%, showing an IC_50_ of 16.70 ± 5.00 μM, similarly to kojic acid (IC_50_ = 16.80 ± 4.60 μM) at a concentration of 200 μM. Furthermore, this compound (**42**) effectively suppressed the melanin production by 60% at a concentration of 15 μM without any effect on the cell viability of B16-F10 melanoma cells. Recently, Qu et al. [53] identified an isoflavone, namely puerarin (daidzein-8-*C*-glucoside) (**43**) (7.66 mg/g), as a major compound in the *Puerariae lobatae* radix. The isoflavone (**43**) effectively inhibited the catalytic oxidation process of the tyrosinase enzyme with an IC_50_ of 0.537 mg/mL in a concentration-dependent manner. In 2018, Wagle et al. [54] described fourteen isoflavonoid derivatives from *Pueraria lobata* (Fabaceae) roots. Among the list of these compounds, calycosin (3′,7-dihydroxy-4′-methoxyisoflavone) (**44**) was characterized as the only potent inhibitor one (inhibition% = 85.60, IC_50_ = 1.45 ± 0.03 μM), superior to the standard, kojic acid (inhibition% = 74.27, IC_50_ = 9.14 ± 0.01 μM). Kim and his team published a report about the isoflavonoids isolated from *Apios americana* (Fabaceae) tubers and screened them for tyrosinase inhibition [55]. Of the evaluated compounds, lupinalbin A (**45**) and 2′-hydroxygenistein-7-*O*-gentibioside (**46**) dose-dependently displayed moderate competitive tyrosinase inhibitors with IC_50_ values of 39.70 ± 1.5 and 50.00 ± 3.70 µg/mL, respectively, whereas the IC_50_ of kojic acid was equal 25.20 ± 0.80 µg/mL. The chemical structures of the isoflavones with anti-tyrosinase activities are presented in Figure 4.

### 3.4. Flavan-3-ols

**Figure 5:**
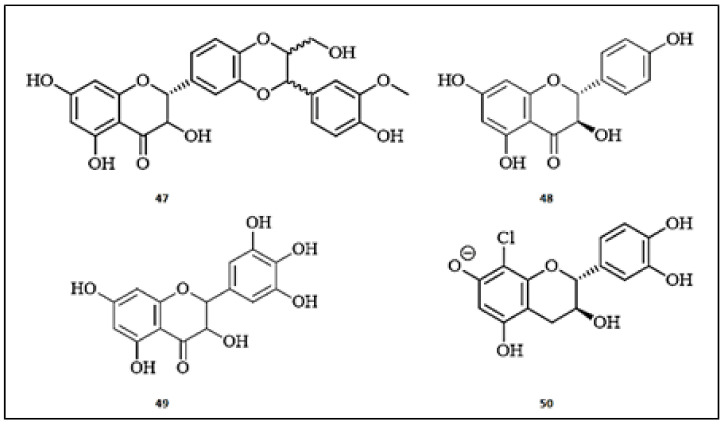
The chemical structures of flavan-3-ols with anti-tyrosinase activity. (**47**) Silybin; (**48**) (+)-Dihydrokaempferol; (**49**) Dihydromyricetin; (**50**) (−)-8-chlorocatechin.

The milk thistle (*Silybum marianum*) is well-known to be rich in flavonolignans with antioxidant, anti-inflammatory, antiviral and antifibrotic properties [56]. Among these compounds, silybin (**47**) was demonstrated to be the most potent one for tyrosinase inhibition, with an IC_50_ value of 1.70 ± 0.07 µM, compared to kojic acid (IC_50_ = 15.30 ± 0.50 µM) [57]. Based on kinetic studies, silybin (**47**) showed mixed type 1 inhibition of enzyme and a significant binding affinity with K_i_ value of 0.7 µM. A study published by Chunhakant and Chaicharoenpong reported characterization of phytochemicals from *Manilkara zapota* (Sapotaceae) bark [58]. Among the isolated compounds, (+)-dihydrokaempferol (**48**) demonstrated the most potent tyrosinase inhibitory effect with IC_50_ of 55.41 ± 0.38 μM, comparable to kojic acid (IC_50_ = 53.43 ± 0.38 μM) and α-arbutin (IC_50_ = 365.93 ± 0.45 μM). Furthermore, (+)-dihydrokaempferol (**48**) exhibited the strongest antioxidant activity of isolated compounds through three different assays; 2,2-diphenyl-1-picrylhydrazyl (DPPH; IC_50_ = 2.21 ± 0.77 μM), 2,2′-azino-bis (3-ethylbenzothiazoline-6-sulfonic acid (ABTS; IC_50_ = 214.83 ± 0.51 μM), and ferric reducing antioxidant power (FRAP; 6.23 ± 0.10 μM), compared with Trolox ((DPPH; IC_50_ = 1.92 ± 0.22 μM, ABTS; IC_50_ = 188.39 ± 0.43 μM and FRAP; 6.10 ± 0.28 μM). Fan et al. [59] studied the molecular docking of dihydromyricetin (**49**), a common natural flavonoid, on tyrosinase. This compound is fitted in the pocket of tyrosinase with hydrophobic interactions and hydrogen bonds, resulting in conformational changes of tyrosinase that hinder substrate binding. Consequently, it inhibited tyrosinase activity in a mixed-type manner with an IC_50_ value of 3.66 ± 0.14 × 10^−5^ mol/L, compared with kojic acid (IC_50_ = 4.64 ± 0.37 × 10^−5^ mol/L). In addition, the combination of dihydromyricetin (**49**) with vitamin D_3_ displayed a synergistic effect on the enzyme inhibition. In Turkey, a group of researchers isolated a new catechin, namely (−)-8-chlorocatechin (**50**), from *Quercus coccifera* bark [60]. The isolated compound (**50**) expressed tyrosinase inhibition with IC_50_ value of 4.05 ± 0.30 µg/mL, more potent even than kojic acid (IC_50_ = 50.75 ± 0.25 µg/mL). Molecular modeling studies revealed the good fitting properties of the compound to the catalytic site of tyrosinase via 4-chromanone moiety with its hydroxyl groups. The chemical structures of the flavan-3-ols with anti-tyrosinase activities are presented in Figure 5.

### 3.5. Flavanones 

**Figure 6:**
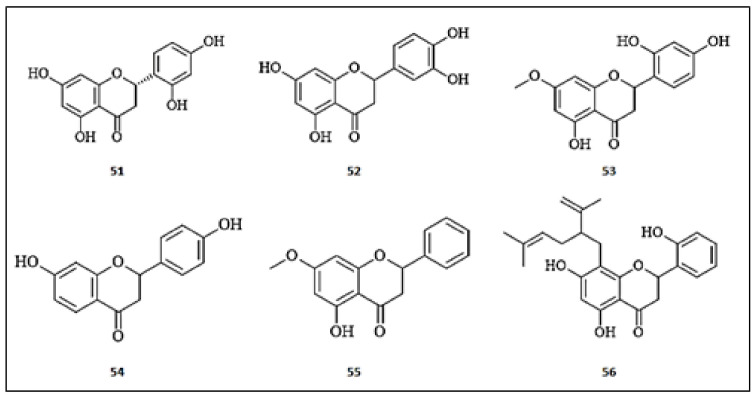
The chemical structures of flavanones with anti-tyrosinase activity. (**51**) Steppogenin; (**52**) Eriodictyol; (**53**) Artocarpanone; (**54**) Liquiritigenin; (**55**) Pinostrobin; (**56**) Kushenol A.

An in depth phytochemical study of 70% ethanolic extract of *Morus alba* leaves resulted in the isolation of twelve flavonoids which were evaluated for anti-melanogenesis effects in B16-F10 mouse melanoma cells [61]. Among these compounds, steppogenin (5,7,2′,4′-tetrahydroxyflavanone) (**51**) dose-dependently inhibited melanin production via the suppression of intracellular tyrosinase activity, MITF and TRP-1. Moreover, steppogenin (**51**) effectively modulated CREB and p38 signaling pathways in alpha-melanocyte stimulating hormone (α-MSH)-activated B16-F10 melanoma cells, proceeding to anti-melanogenesis effects. Interestingly, the structure–activity relationship elucidated that the compound possesses a unique structure for tyrosinase inhibition, including hydroxyl groups at C-2′ and C-4′ of the B-ring, at C-5 and C-7 of the A-ring and free C-3. Eriodictyol (3′,4′,5,7-tetrahydroxyflavanone) (**52**), a flavanone isolated from *Eriodictyon californicum*, has been investigated for anti-melanogenic effects in cultured murine melanoma cells (B16-F10) and primary human keratinocytes (PHK) [62]. Steppogenin (5,7,2′,4′-tetrahydroxyflavanone) (**51**) displayed time and concentration-dependent inhibition of tyrosinase in the tested cells. Moreover, it exerted intracellular free radical scavenging potential against hydrogen peroxide (H_2_O_2_)-induced oxidative stress at a concentration of 50 μM in B16-F10 and PHK cells. Among the flavanones isolated from the wood of *Artocarpus heterophyllous,* artocarpanone (2′,4′,5-trihydroxy-7-methoxy-isoflavone) (**53**) demonstrated the most potent inhibitory activity against the tyrosine enzyme with an IC_50_ of 2.00 ± 0.1 μM [34]. Additionally, steppogenin (5,7,2′,4′-tetrahydroxyflavanone) (**51**) and liquiritigenin (4′,7-dihydroxyflavanone) (**54**) demonstrated concentration-dependent inhibition of the enzyme with IC_50_ values of 7.50 ± 0.50 and 22.00 ± 2.50 μM, respectively. They demonstrated more potent inhibition compared to the positive control, kojic acid, with an IC_50_ of 44.60 ± 0.40 μM. Pinostrobin (**55**) was isolated from Egyptian *Propolis* among other phenolic compounds and was evaluated for its tyrosinase inhibitory activities [63]. Pinostrobin ((2s)-5-hydroxy-7-methoxyflavanone) (**55**) was the most potent of the isolated compounds of *Propolis*, demonstrating a moderate inhibitory activity (36.30%) compared with vitamin C, demonstrating 60% inhibition. It was concluded that flavonoids possessing hydroxyl groups at the A and B rings are crucial tyrosinase inhibitors via Cu^2+^ chelate formation. Investigating the tyrosine inhibitory activity of the isolated flavonoids of *Sophora flavescens,* kushenol A (**56**) demonstrated the most potent inhibitory activity, demonstrating an IC_50_ value of 1.10 ± 0.70 μM [64]. Molecular docking analysis coupled with enzyme kinetics supported the significant inhibitory activity, demonstrating hydrogen bonding with the Gly326, Gln67, Lys70, and Tyr78 residues of tyrosinase. The chemical structures of the flavanones with anti-tyrosinase activities are presented in Figure 6.

### 3.6. Chalcones 

Ticona et al. [29] identified four anti-tyrosinase chalcones from a dichloromethane extract of *Loranthus acutifolius*’s aerial parts. These compounds were identified as 2′4′-dihydroxy-6′-methoxy-chalcone (**57**) (IC_50_ = 5.70 ± 0.02 μM), comparable with kojic acid (IC_50_ = 13.90 ± 0.34 μM). Among seventeen phenolic compounds isolated from *Angelica keiskei* (Umbelliferae) roots, Lee et al. [65] found that the chalcone compound, xanthoangelol (2′,4,4′-trihydroxy-3′-geranylchalcone) (**58**), showed a moderate tyrosinase inhibitory property with IC_50_ values of 15.87 ± 1.21 μM, compared to kojic acid (IC_50_ = 3.80 ± 0.20 μM). Interestingly, this study used a combinatorial novel approach that includes in vitro tyrosinase inhibition assay coupled with UPLC-MS/MS to screen isolated compounds. Flavokawain A (2′-hydroxy-4,4′,6′-trimethoxychalcone) (**59**) and flavokawain B (4′,6′-Dimethoxy-2′-hydroxychalcone) (**60**), naturally occurring chalcones of Kava (*Piper methysticum*), were investigated for their melanogenic inhibition in α-MSH-induced B16/F10 cells and zebrafish [66]. Both compounds suppressed specific cellular tyrosinase activity by 7- and 9-fold, respectively, without any toxic effects in zebrafish. Mechanistically, they downregulated MITF expression, which in turn down-regulates TYR, TRP-1, and TRP-2. Five chalcones were isolated from *Humulus lupulus* through tyrosinase activity-guided fractionation of its methanol extract [67]. Among the isolated chalcones, xanthohumol (2′,4,4′-trihydroxy-6′-methoxy-3′-prenylchalcone) (**61**), xanthoumol B (dehydrocycloxanthohumol Hydrate) (**62**) and xanthohumol C (dehydrocycloxanthohumol) (**63**) demonstrated dose-dependent inhibition of the monophenolase and diphenolase activities of tyrosinase, with IC_50_ values ranging from from 15.40 to 22.1 μM and from 41.10 to 46.70 μM, respectively. The more potent inhibition was demonstrated with chalcones bearing an isoprenyl group at ring A. Kinetic studies using Dixon and Lineweaver–Burk plots demonstrated the competitive inhibitory activity of the isolated chalcones against tyrosinase.

**Figure 7:**
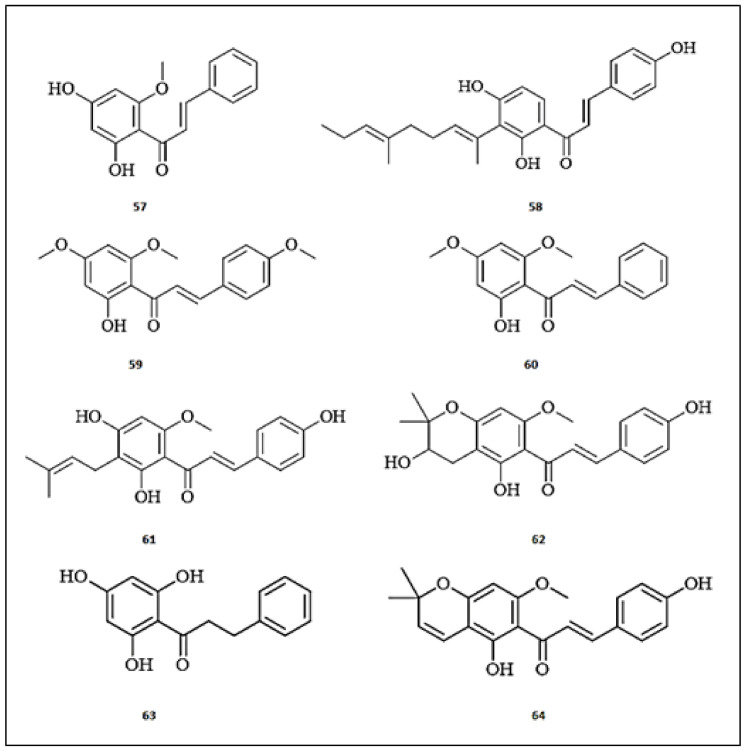
The chemical structures of chalcones with anti-tyrosinase activity. (**57**) 2′4′-dihydroxy-6′-methoxy-chalcone; (**58**) Xanthoangelol; (**59**) Flavokawain A; (**60**) Flavokawain B; (**61**) Xanthohumol; (**62**) Xanthoumol B; (**63**) Xanthohumol C; (**64**) 2′,4′,6′-trihydroxydihydrochalcone.

The methanolic extract of *Greyia radlkoferi* and its isolated flavonoids was investigated for their tyrosinase inhibitory effects [68]. The extract displayed potent inhibitory activity against tyrosinase with IC_50_ value of 17.96 μg/mL. Among the isolated flavonoids, 2′,4′,6′-trihydroxydihydrochalcone (**64**) was characterized as the most potent tyrosinase inhibitor, with an IC_50_ value of 17.70 μg/mL, comparable to that of kojic acid (IC_50_ = 3.87 μg/mL). The investigation of the underlying mechanism of inhibition using RT-qPCR was demonstrated to be post-transcriptional. Moreover, molecular docking studies revealed the influence of interaction with Cu^2+^ ions at the active site on tyrosinase. The chemical structures of the chalcones with anti-tyrosinase activities are presented in Figure 7.

### 3.7. Prenylated Flavonoids 

Three new isoprenylated flavonoids, namely, kuwanon J (**65**), sanggenon C (**66**), and sanggenon M (**67**) with sanggenon O (**68**) were isolated from the petroleum ether extract of *Morus nigra* twigs [69]. All isolated compounds showed a significant inhibitory effect on tyrosinase, with kuwanon J (**65**) being the most potent, with IC_50_ values of 0.17 ± 0.01 µM, followed by sanggenon O (**68**) (IC_50_ = 1.15 ± 0.03 µM), sanggenon C (**66**) (IC_50_ = 1.17 ± 0.03 µM), then sanggenon M (**67**) (IC_50_ = 13.06 ± 0.58 µM), compared to kojic acid (IC_50_ = 32.62 ± 1.24 μM). Two major prenylated flavonoids, namely kuwanon O (**69**) and sanggenon T (**70**), were isolated from *Morus australis* root extract and evaluated for depigmenting effects in different melanocyte systems and artificial skin models [70]. Both compounds exhibited significant whitening effects in both murine b16 and melan-a cell lines via the induction of MITF posttranscriptional degradation without the downregulation of mRNA expression, and consequently a remark-reduced production of TRP-1 and TRP-2 occurred in murine b16 cell line. Meanwhile, tyrosinase was inhibited in melan-a cells at both the transcription and translation levels, with kuwanon O being the most active compound. Interestingly, this study deduced the structure relationship between the necessity of the isoprene group and potent hypopigmenting effect. Moreover, the prenylated flavonoids, kuwanon O (**69**) and sanggenon T (**70**), demonstrated an outstanding depigmenting effect in the artificial skin model. Dehydroglyasperin C (**71**), a major prenylflavonoid of *Glycyrrhiza uralensis* (Fabaceae), suppressed intracellular tyrosinase activity and related proteins (TYR-1 and TRP-2) at a low concentration (1 μM) in α-MSH-induced B16F1 melanoma cells [71]. Furthermore, it significantly downregulated MITF through the activation of extracellular signal-regulated kinase (ERK) phosphorylation and the suppression of cAMP-CREB signaling pathway.

Another study was conducted on kazinol U (**72**), a prenylated form of *Broussonetia kazinoki* Sieb bark (Moraceae), to evaluate its anti-melanogenic effect on human melanoma cells, normal human melanocytes, and zebrafish [72]. Kazinol U dose-dependently inhibited intracellular tyrosinase activity, the expression of its related proteins (TYR-1 and 2), the basal and IBMX-induced tyrosinase promoter activity in B16F10 cells, compared with arbutin, a standard hypopigmenting drug. Moreover, kazinol U (**72**) potently downregulated MITF by means of the acceleration of AMPK and MAPK protein phosphorylation, which are MITF inhibitors. Moreover, it exhibited anti-melanogenic effects in an vivo model of zebrafish without developmental defects. Cycloheterophyllin (**73**), a prenylated flavonoid isolated from *Artocarpus lowii* heartwood and leaves (Moraceae), was characterized as a tyrosinase inhibitor (IC_50_ = 104.6 μM), compared to kojic acid (IC_50_ = 219.6 μM) [66]. Furthermore, it showed potent antioxidant effects in the DPPH assay (SC_50_ = 102.80 μM), FRAP method (4.70 mM) and ABTS (SC_50_ = 320.00 μM) [73].

**Figure 8:**
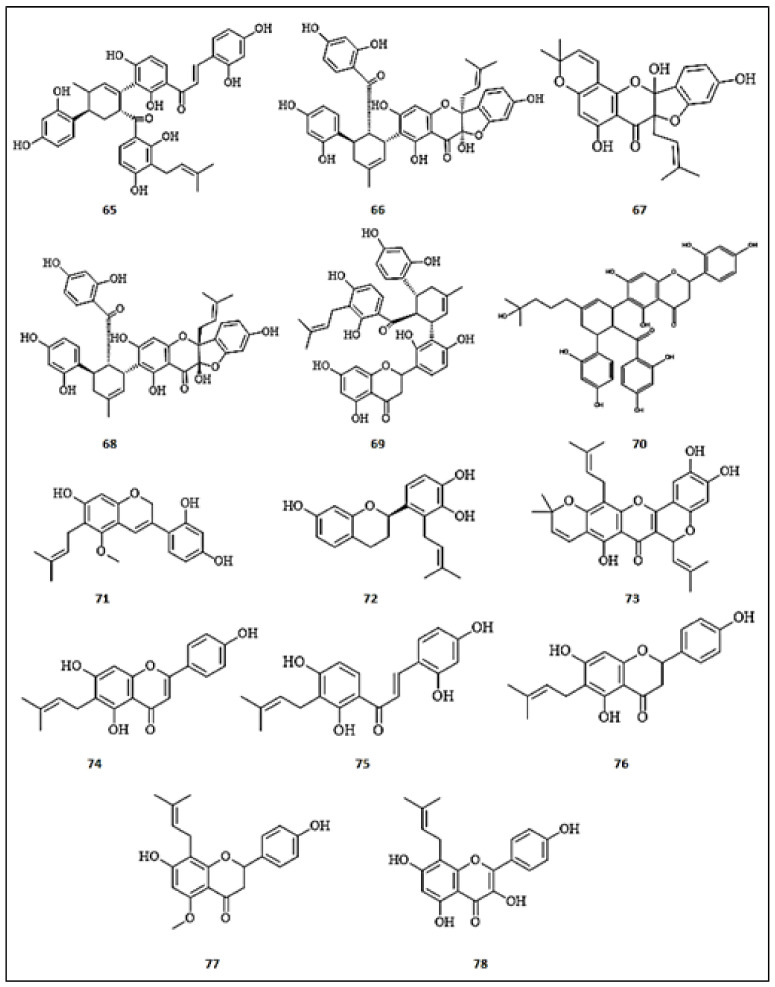
Chemical structures of prenylated flavonoids with anti-tyrosinase activity. (**65**) Kuwanon J; (**66**) Sanggenon C; (**67**) Sanggenon M; (**68**) Sanggenon O; (**69**) Kuwanon O; (**70**) Sanggenon T; (**71**) Dehydroglyasperin C; (**72**) Kazinol U; (**73**) Cycloheterophyllin; (**74**) Morachalcone A; (**75**) 6-prenylapigenin; (**76**) 6-Prenylnaringenin; (**77**) Isoxanthohumol; (**78**) 8-Prenylkaempferol.

Upon screening analysis of nine prenylated phenolic compounds isolated from *Artocarpus pithecogallus* (Moraceae) twigs, morachalcone A (**74**) and 6-prenylapigenin (**75**) exhibited the most potent tyrosinase effects with IC_50_ values of 0.77 ± 0.01 μM and 24.29 ± 0.12 μM, respectively, compared to kojic acid (IC_50_ = 17.32 ± 0.24 μM) [74]. Kim et al. [69] isolated two flavanones, 6-prenylnaringenin (**76**) and isoxanthohumol (**77**), from *Humulus lupulus* methanol extract, among other flavonoids. Both compounds demonstrated significant inhibition of tyrosinase, with IC_50_ values of 38.1 and 77.4 µM, respectively, for monophenolase and IC_50_ values of 77.2 and 157.4 µM, respectively, for diphenolase. Kinetic studies using Dixon and Lineweaver–Burk plots demonstrated mixed type inhibition by both flavanones against monophenolase. However, noncompetitive inhibition was demonstrated by both compounds against diphenolase. A study by Kim et al. [66] led to the isolation of five flavonoids from the methanol extract of *Sophora flavescents.* Investigating the inhibitory effects of the isolated flavonoids, 8-prenylkaempferol (**78**) demonstrated potent inhibitory activity (IC_50_ = 2.40 ± 1.10 μM) against tyrosinase exceeding the chalcones isolated. Molecular docking simulation revealed the competitive inhibition of the prenylated flavonol via its docking to His244 residue of tyrosinase. The chemical structures of the prenylated flavonoids with anti-tyrosinase activities are presented in Figure 8.

### 3.8. Biflavonoids 

**Figure 9:**
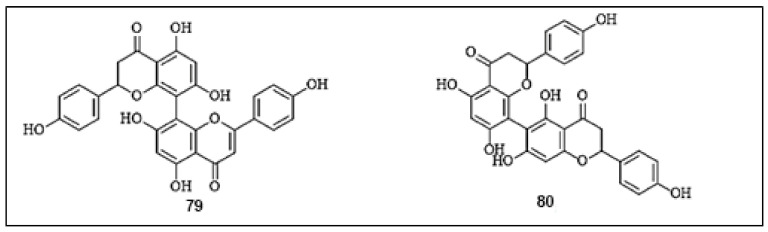
The chemical structures of biflavonoids with anti-tyrosinase activity. (**79**) Rhusflavanone; (**80**) Mesuaferrone B.

Rhusflavanone (**79**) and mesuaferrone B (**80**) are major bioflavonoids, isolated from the methanolic extract of *Mesua ferrea* L. stamens with contents of 0.35 ± 0.04% and 0.55 ± 0.06%, respectively [75]. Both biflavonoids efficiently displayed inhibitory activities against mushroom tyrosinase, with IC_50_ values of 10.6 and 10.3 µg/mL, respectively, compared with arbutin (IC_50_ = 87.20 µg/mL). Furthermore, the dimerization of flavonoid units was found to contribute effectively in potent tyrosinase inhibitory activities, comparable to the original flavonoid monomers. The chemical structures of the biflavonoids with anti-tyrosinase activities are presented in Figure 9.

We summarize the promising antityrosinase flavonoids, their sources, the type of the performed assays and their significance in Table 1. Additionally, we illustrated the different modes of actions reported for tested flavonoids in Table 2. As observed, flavonoids exhibited antityrosinase activity through different pathways such as competitive inhibition, non-competitive inhibition, mixed inhibition of tyrosinase, downregulation of MITF expression, and suppression of the cAMP-CREB signaling pathway.

## 4. Conclusions and Future Perspectives

In this review, reports from 2016–2021 on recently isolated flavonoids with tyrosinase inhibitor activity were summarized and critically analyzed. The review focused on the potential activities of different categories of flavonoids, including flavones, flavonols, isoflavones, flavan-3-ols, flavanones, prenylated flavonoids and biflavonoids in modulating the activity of tyrosinase enzyme. The studies varied between in vitro, in vivo and in silico computational assays. Moreover, the integration of inhibition kinetics and docking studies aided in the better understanding of structure–activity relationship (SAR) of flavonoids and tyrosinase inhibitory activity. It is worth mentioning that the number and the position of hydroxyl groups especially at ring B drastically affect the activity of different flavonoid classes via Cu^2+^ chelate formation. In addition, the isoprene moiety and the dimerization of flavonoids contribute in the inhibitory activity of prenylated flavonoids and biflavonoids, respectively. Additionally, the mechanisms of tyrosinase activity inhibition for some promising flavonoids were discussed. Several inhibition mechanisms have been reported for the described inhibitors, pointing to the copper chelating and/or hydrophobic moieties as key structural requirements to achieve good inhibition properties. Some flavonoids were proved to suppress the expression of tyrosinase via the modulation of certain signaling pathways such as MITF, AMPK and MAPK proteins phosphorylation, CREB and p38.

Despite this extensive research, we observed that the studies addressing the safety profile the promising flavonoids are very scarce. Additionally, some studies are recommended to be extrapolated from in vitro assays to animal and clinical studies to assess the pharmacokinetics, topical permeation, bioavailability, dose issues, lifespan, anatomy and metabolic status. As the conventional whitening market products suffer from severe side effects such as dermatitis and skin cancer in long-term use, we highly recommend well-designed long-term studies in the human subjects to investigate the potential toxic effects, biochemical and molecular mechanisms of the bioactive flavonoids. Interestingly, these studies will potentially push researchers to develop a novel drug for treating skin-aging ailments using medicinal chemistry approaches to synthesize more potent derivatives and study their detailed mechanisms of action.

## Figures and Tables

**Table 1:** List of promising antityrosinase flavonoids, their source, the type of the performed assays and their significance.

Active Flavonoid		Source	Assay Type	IC_50_ or % Inhibition	Ref.
***Flavones***					
1.	Tricin (3′,5′-dimethoxy-4′,5,7-trihydroxyflavone)	*Oryza sativa*	In vitro	15.69%	[17]
2.	Luteolin-7-sulfate (3′,4′,5-trihydroxy-flavone-7-sulphate)	*Phyllospadix iwatensis* *Zostera marina*	In vitro	Dose-dependent	[18]
3.	2′,4′,6-trimethoxyflavone	*Loranthus acutifolius*	In vitro	4.00 μM	[20]
4.	3′,4′,5-trihydroxy-6,7,8-trimethoxyflavone			11.30 μM	
5.	4′,5-dihydroxy-6,7,8-trimethoxyflavone			8.60 μM	
6.	Baicalein (5,6,7-trihydroxyflavone)	*Oroxylum indicum*	In silico	0.29 mM	[21]
7.	Oroxin A (baicalin-7-glucoside)	*Oroxylum indicum*	In silico	0.50 mM	[22]
8.	Isovitexin (apigenin-6-C-glucoside)	*Achillea alpina*	In vitro	Dose-dependent	
9.	Diosmetin (3′,5,7-trihydroxy-4′-methoxyflavone)	*Trichosanthes kirilowii*	In vitro	Dose-dependent	[23]
10.	Chrysoeriol (4′,5,7-trihydroxy-3′-methoxyflavone)				
11.	Scutellarin (4′,5,6,7-tetrahydroxy-flavone-7-*β*-d-glucopyranuronoside)				
12.	3′,5-dihydroxy-7-(*β*-d-glucopyranosyloxy)−4′-methoxyflavone				
13.	Sulfuretin (6,3′,4′-trihydroxyaurone)	*Rhus verniciflua*	In vivo	20 μM	[24]
14.	artocaepin E	*Artocarpus heterophyllous*	In vitro	6.7 μM	[25]
15.	Genkwanin (4′,5-dihydroxy-7-methoxyflavone)	*Daphne gnidium* *Alnus glutinosa* *Asplenium normale*	In vitro	Dose-dependent	[26]
16.	Apigenin-7-*O*-*β*-d-glucopyranoside	*Thymus* species	In vitro	Dose-dependent	[26]
17.	Swertiajaponin ((6-C-*β*-d-glucopyranosyl-7-*O*-methylluteolin)	*Swertia japonica, Cymbopogon citratus*	Human skin model	43.47 μM	[27]
18.	Norartocarpetin (5,7,2′,4′-tetrahydroxyflavone)	*Artocarpus dadah*	In silico	0.12 μM	[28]
19.	Luteolin (3′,4′,5,7-tetrahydroxy-flavone)	*Reseda luteola* *Elsholtzia rugulosa*		266.67 μM	
20.	Hypolaetin-7-*O*-*β*-d-glucopyranoside (8-Hydroxyluteolin-7-*O*-*β*-d-glucopyranoside)	*Juniperus chinensis*	In vitro In silico	73.30%	[29]
***Flavonols***					
21.	Swertiajaponin ((6-C-*β*-d-glucopyranosyl-7-*O*-methylluteolin)	*Swertia japonica* *Cymbopogon citratus*	Human skin model	Dose-dependent	[27]
22.	4′,5,6,7-tetramethoxyflavone				
23.	Nobiletin				
24.	3,3′,4′,5,6,7-hexamethoxyflavone				
25.	Hyperoside	*Rosa rugosa*	In vitro	IC_50_ ˂ 1 μg/mL	[32]
26.	Kaempferol-3-*O*-rutinoside				
27.	Rutin (quercetin 3-rutinoside)	*Rosa rugose* *Myrsine africana*	In vitro	2.30 μM	[32,33]
28.	Myricetin-3-*O*-*α*-l-rhamnopyranoside	*Myrsine africana*	In vitro In silico	2.00 μM	[31,41]
29.	Myricetin-3-*O*-*β*-galactopyranoside	*Limonium tetragonum*	In vitro	65%	[34]
30.	Quercetin-3-*O*-*β*-galactopyranoside			63%	
31.	5,7,4′,5′-tetrahydoxyflavonol 2′-[propanoic acid-(2″’-acetoxy-1″’-sulfonyl)]−5′-*O*-*β*-d-glucopyranoside	*Cotula anthemoides*	In vitro	100 ± 0.5 μM	[36]
32.	Quercetin	*Persicaria senticosa*	In silico In vitro	3.08 ± 0.74 mol L^−1^ 14.31 ± 3.93 μM	[37,38]
33.	Quercetin-3-*O*-*α*-arabinopyranosyl-(1→6)-*β*-glucopyranoside	*Scrophularia lucida*	In silico	46.94 ± 3.09	[39]
34.	Hesperidin (hesperetin 7-rutinoside)			Undetermined	
35.	8-*β*-d-glucopyranosyloxy-3′,4′,5,5′-tetrahydroxy-7-methoxy-3-*α*-l-rhamnopyranosyloxyflavone	*Atraphaxis frutescens*	In vitro	0.90 ± 0.02 mM	[40]
36.	3′,4′,5,5′,8-pentahydroxy-7-methoxy-3-*α*-l-rhamnopyranosyloxyflavone			1.20 ± 0.03 mM	
37.	Europetin 3-*O*-*α*-l-rhamnopyranoside			1.10 ± 0.07 mM	
38.	Myricetin-3-*O*-*α*-l-rhamnopyranoside	*Myrsine africana*	In vitro In silico	0.12 ± 0.002 mM	[41]
***Isoflavones***					
39.	Glabridin	*Glycyrrhiza glabra*	In silico	0.43 μmol/L	[41]
40.	Neobavaisoflavone	*Pueraria 1lobate*	In vitro Human skin model	10–45%	[42,43]
41.	(6aR,11aR)-3,8-dihydroxy-9-methoxy pterocarpan	*Dalbergia parviflora*	In vitro	84.60%	[45]
42.	Puerarin (daidzein-8-*C*-glucoside)	*Pueraria lobata*	In vitro	0.537 mg/mL	[46]
43.	Calycosin (3′,7-dihydroxy-4′-methoxyisoflavone)		In vitro In silico	85.60%	[47]
44.	Lupinalbin A	*Apios americana*	In vitro In silico	39.70 ± 1.5 µg/mL	[48]
45.	2′-hydroxygenistein-7-*O*-gentibioside			50.00 ± 3.70 µg/mL	
***Flavan-3-ols***					
46.	Silybin	*Silybum marianum*	In vitro	1.70 ± 0.07 µM	[50]
47.	(+)-dihydrokaempferol	*Manilkara zapota*	In vitro	45.35 ± 0.60 µM	[51]
48.	Dihydromyricetin		In silico	36.6 ± 0.14 µM	[52]
49.	(−)-8-chlorocatechin	*Quercus coccifera*	In silico	4.05 ± 0.30 µg/mL	[53]
***Flavanones***					
50.	Steppogenin (5,7,2′,4′-tetrahydroxyflavanone)	*Artocarpus heterophyllous* *Morus alba*	In vitro In silico	7.50 ± 0.50 μM	[25,54]
51.	Eriodictyol (3′,4′,5,7-tetrahydroxyflavanone)	*Eriodictyon californicum*	In vitro	Dose-dependent	[55]
52.	Artocarpanone (2′,4′,5-trihydroxy-7-methoxy-Isoflavone)	*Artocarpus heterophyllous*	In vitro	2.0 ± 0.1 μM	[25]
53.	Liquiritigenin (4′,7-dihydroxyflavanone)	*Artocarpus heterophyllous*	In vitro	22.00 ± 2.50	[25]
54.	Pinostrobin ((2s)-5-hydroxy-7-methoxyflavanone)	Egyptian *Propolis*		36.30%	
55.	kushenol A	*Sophora flavescens*	In silico	1.10 ± 0.70 μM	[57]
***Chalcones***					
56.	2′4′-dihydroxy-6′-methoxy-chalcone	*Loranthus acutifolius*		IC_50_ = 5.70 ± 0.02 μM	[56]
57.	Xanthoangelol (2′,4,4′-trihydroxy-3′-geranylchalcone)	*Angelica keiskei*	In vitro	15.87 ± 1.21 μM	[58]
58.	Flavokawain A (2′-hydroxy-4,4′,6′-trimethoxychalcone)	*Piper methysticum*	In vitro In vivo zebrafish	Dose-dependent	[59]
59.	Flavokawain B (4′,6′-dimethoxy-2′-hydroxychalcone)				
60.	Xanthohumol (2′,4,4′-trihydroxy-6′-methoxy-3′-prenylchalcone)	*Humulus lupulus*	In vitro	15.40–22.1 μM	[60]
61.	Xanthoumol B (dehydrocycloxanthohumol hydrate)			41.10–46.70 μM	
62.	Xanthoumol C (dehydrocycloxanthohumol)				
63.	2′,4′,6′-trihydroxydihydrochalcone	*Greyia radlkoferi*	In vitro In silico	17.70 μg/mL	[61]
***Prenylated flavonoids***					
64.	Kuwanon J	*Morus nigra*	In vitro	0.17 ± 0.01 µM	[62]
65.	Sanggenon C			1.17 ± 0.03 µM	
66.	Sanggenon M			13.06 ± 0.58 µM	
67.	Sanggenon O			1.15 ± 0.03 µM	
68.	Kuwanon O	*Morus australis*	In vitro Artificial skin models	15-20% 8-12%	[63]
69.	Sanggenon T				
70.	Dehydroglyasperin C	*Glycyrrhiza uralensis*	In vitro	Dose-dependent	[64]
71.	Kazinol U	*Broussonetia kazinoki* Sieb	In vitro In vivo zebrafish	Dose-dependent	[65]
72.	Cycloheterophyllin	*Artocarpus lowii*	In vitro	104.6 μM	[66]
73.	Morachalcone A	*Artocarpus pithecogallus*	In vitro	0.77 ± 0.01 μM	[67]
74.	6-prenylapigenin			24.29 ± 0.12 μM	
75.	6-prenylnaringenin	*Humulus lupulus*	In vitro	38.1 µM	[60]
76.	Isoxanthohumol			77.4 µM	
77.	8-prenylkaempferol	*Sophora flavescents*	In silico	2.40 ± 1.10 μM	[57]
***Biflavonoids***					
78.	Rhusflavanone	*Mesua ferrea*	In vitro	10.60 µg/mL	[68]
79.	mesuaferrone B			10.30 µg/mL	

**Table 2:** Summarized modes of tyrosinase inhibitory actions for flavonoids.

Mode of Action	Compounds
**Competitive inhibition of tyrosinase**	2′,4′,6-trimethoxyflavone (**3**) 3′,4′,5-trihydroxy-6,7,8-trimethoxyflavone (**4**) 4′,5-dihydroxy-6,7,8-trimethoxyflavone (**5**) Artocaepin E (**14**) Hypolaetin-7-*O*-*β*-d-glucopyranoside (**20**) Quercetin-7-*O*-*α*-l-rhamnopyranoside (**21**) Lupinalbin A (**45**) 2′-hydroxygenistein-7-*O*-gentibioside (**46**) (+)-dihydrokaempferol (**48**) (−)-8-chlorocatechin (**50**) Xanthohumol (**61**) Xanthoumol B (**62**) Xanthoumol C (**63**) 8-prenylkaempferol (**78**)
**Non-competitive inhibition of tyrosinase**	Tricin (**1**) Luteolin (**11**) Glabridin (**40**)
**Mixed** **inhibition of tyrosinase**	Puerarin (**43**) Silybin (**47**) Dihydromyricetin (**49**) 6-prenylnaringenin (**76**)
**Downregulation of MITF expression**	Luteolin-7-sulfate (**2**) Sulfuretin (**13**) Swertiajaponin (**17**) Neobavaisoflavone (**41**) Flavokawain A (**59**) Dehydroglyasperin C (**71**) Kazinol U (**72**)
**Suppression of cAMP-CREB signaling pathway**	Luteolin-7-sulfate (**2**) Steppogenin (**51**) Dehydroglyasperin C (**71**)

## Data Availability

Not applicable.
